# deepBase v2.0: identification, expression, evolution and function of small RNAs, LncRNAs and circular RNAs from deep-sequencing data

**DOI:** 10.1093/nar/gkv1273

**Published:** 2015-11-20

**Authors:** Ling-Ling Zheng, Jun-Hao Li, Jie Wu, Wen-Ju Sun, Shun Liu, Ze-Lin Wang, Hui Zhou, Jian-Hua Yang, Liang-Hu Qu

**Affiliations:** RNA Information Center, Key Laboratory of Gene Engineering of the Ministry of Education, State Key Laboratory for Biocontrol, Sun Yat-sen University, Guangzhou 510275, P.R. China

## Abstract

Small non-coding RNAs (e.g. miRNAs) and long non-coding RNAs (e.g. lincRNAs and circRNAs) are emerging as key regulators of various cellular processes. However, only a very small fraction of these enigmatic RNAs have been well functionally characterized. In this study, we describe deepBase v2.0 (http://biocenter.sysu.edu.cn/deepBase/), an updated platform, to decode evolution, expression patterns and functions of diverse ncRNAs across 19 species. deepBase v2.0 has been updated to provide the most comprehensive collection of ncRNA-derived small RNAs generated from 588 sRNA-Seq datasets. Moreover, we developed a pipeline named lncSeeker to identify 176 680 high-confidence lncRNAs from 14 species. Temporal and spatial expression patterns of various ncRNAs were profiled. We identified approximately 24 280 primate-specific, 5193 rodent-specific lncRNAs, and 55 highly conserved lncRNA orthologs between human and zebrafish. We annotated 14 867 human circRNAs, 1260 of which are orthologous to mouse circRNAs. By combining expression profiles and functional genomic annotations, we developed lncFunction web-server to predict the function of lncRNAs based on protein-lncRNA co-expression networks. This study is expected to provide considerable resources to facilitate future experimental studies and to uncover ncRNA functions.

## INTRODUCTION

Eukaryotic genomes encode thousands of small and large non-coding RNAs (ncRNAs) (1,2), such as microRNAs (miRNAs) (3), long non-coding RNAs (lncRNAs) (1,2) and circular RNAs (circRNAs) (4). In contrast to the well-known miRNAs, which have been found to be key players in various biological processes, the functions of the majority of lncRNAs and circRNAs have not been fully investigated (1,2).

To better understand the functions of these ncRNAs, a systematic catalog of ncRNA transcripts and their expressions across tissues and evolutional conservation across species is necessary (1,2). To date, many databases have been developed to curate computationally predicted and experimentally verified ncRNAs, such as LncRNAdb (5), GENCODE (6), lncRNAtor (7), ChIPBase (8), NONCODE (9), miRBase (10), starBase (11) and circBase (12). However, these databases either focus on limited species or contain insufficient expression or evolution data.

Recent advances in high-throughput next-sequencing technology have produced large numbers of short and long RNA sequences, and enable the detection and profiling of known and novel ncRNAs at unprecedented sensitivity and depth (1). Many studies have identified thousands of sRNAs, lncRNAs and circRNAs from deep-sequencing datasets in various species, including human (13–17), mouse (16,18), fruitfly (19), nematode (16,20) and zebrafish (21,22). With the increasing amount of deep-sequencing data available, there is a great need to integrate these large-scale data sets to explore the expression, evolution and function of diverse ncRNAs.

**Figure 1.**
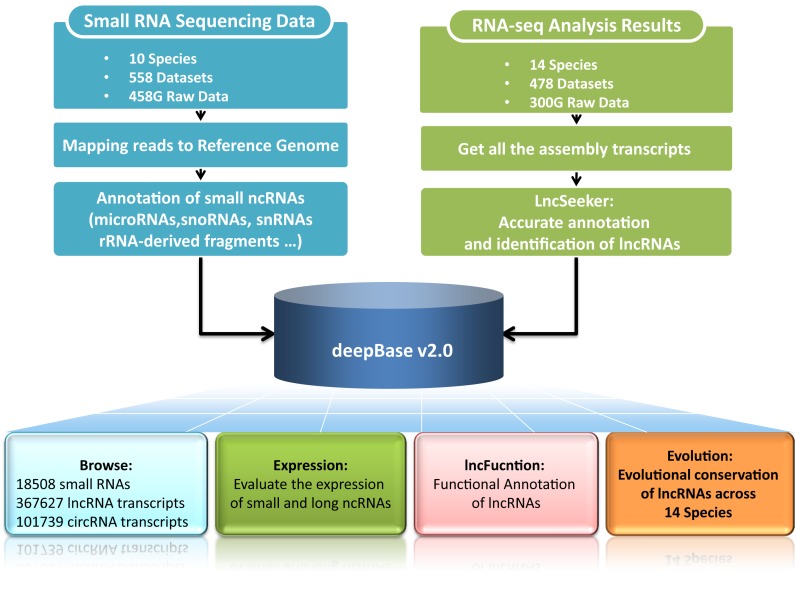
A system-level overview of the deepBase v2.0 core framework. A total of 558 small RNA datasets and 478 RNA-seq datasets were retrieved from NCBI GEO or SRA database. All the small and large noncoding RNAs were identified. The expression, evolution and functions of these ncRNAs were further analyzed. All the results generated by deepBase v2.0 were deposited in MySQL relational databases and displayed in the visual browser and web pages.

To meet above-mentioned needs, we have updated deepBase (23) to version 2.0 (deepBase v2.0) (Figure 1). The deepBase v2.0 facilitates the integrative, interactive and versatile display of, as well as the comprehensive annotation and discovery of sRNAs, lncRNAs and circRNAs (Figure 1). In deepBase v2.0, we constructed the most comprehensive expression profiles and evolutional patterns of diverse ncRNAs.

## MATERIALS AND METHODS

### Integration of public small RNA-Seq and RNA-Seq datasets

A total of 558 small RNA deep-sequencing datasets and 478 RNA-seq datasets were compiled in deepBase v2.0 (Table 1). The raw data were downloaded from NCBI GEO and SRA databases (24). All sequencing datasets and their detailed information are listed in Supplementary Table S1. The genome sequences and transcript sequences were downloaded from NCBI Reference Sequences (RefSeq) (25), UCSC Bioinformatics websites (26) and the following specialized databases: human (UCSC hg19), mouse (UCSC mm10), chicken (Gallus gallus, v4) and *Ciona intestinalis* (JGI v2.0) genome sequences were downloaded from the UCSC Bioinformatics website. Chimp (*Pan troglodytes*, panTro4), Gorilla (*Gorilla gorilla gorilla*, gorGor3), Rhesus (*Macaca mulatta*, rheMac3), Rat (*Rattus norvegicus*, rn6), Cow (*Bos taurus*, bosTau7), Opossum (*Monodelphis domestica*, monDom5), Platypus (*Ornithorhynchus anatinus*, ornAna1), X. tropicalis (*Xenopus tropicalis*, xenTro3), Zebrafish (*Danio rerio*, danRer7) and C. elegans (*Caenorhabditis elegans*, ce10) genome sequences were downloaded from the UCSC Bioinformatics website. The *Caenorhabditis remanei* and *Caenorhabditis briggsae* genome sequences were downloaded from WormBase (27), the *Drosophila melanogaster* genome sequences were download from Flybase (28). The *Bombyx mori* genome sequences were download from silkDB (29). All known miRNAs were downloaded from miRBase (release 21) (30). Other ncRNAs were downloaded from Ensembl Gene Release 76.

**Table 1.** The datasets that are incorporated into deepBase v2.0

Species	RNA-seq	sRNA-seq	LncRNA	smallRNA	circRNA
Human	82	381	18 964	5008	14 867
Chimpanzee	27	–	13 604	–	–
Gorilla	11	–	13 764	–	–
Rhesus	40	–	31 950	–	–
Mouse	74	128	19 368	3134	1891
Rat	27	–	23 052	–	–
Cow	27	–	16 735	–	–
Pig	–	6	–	812	–
Opossum	19	–	7680	–	–
Platypus	18	–	6621	–	–
Chicken	75	4	19 105	692	–
Xenopus	12	–	3172	–	–
Zebrafish	1	–	851	–	–
C. intestinalis	–	4	–	287	–
Worm	–	3	–	295	–
Drosophila	30	8	1644	1190	–
C. elegans	35	16	170	6763	638
C. briggsae	–	4	–	170	–
C. remanei	–	4	–	157	–

These statistics show the numbers of sequencing experiments (sRNA-Seq and RNA-Seq), number of lncRNAs, number of circRNAs and number of small RNAs identified from sequencing datasets.

### Annotation and Identification of sRNAs, lncRNAs and circRNAs

The raw data downloaded from NCBI SRA or GEO databases were classified into different clades, species, tissues and cell-lines according to the description on the website or related literature. For small RNA annotation, after remove the 3′ adapter sequences, the clean reads were then mapped back to their corresponding reference genome using bowtie program. Bedtools intersect tool was used to inspect how many reads could matched to the region of annotated small RNA genes. The reads which were perfectly located in the annotated gene regions were used for expression evaluation.

To accurately annotate lncRNA, we proposed a pipeline to process transcriptome annotation profiles generated by RNA-seq (usually assembled by Tophat (31) and Cufflinks (32)) using several filters described as follow:

1. Transcript length filter: Multi-exonic transcripts whose length exceeded 200nt were kept.
2. Known non-lincRNA annotation filter: Transcripts were excluded from further consideration if they overlap, or reside within 50nt upstream or downstream of, any exon of any transcript from the following annotation sets: coding genes annotated in RefSeq, Ensembl or UCSC knownGene; pseudogenes annotated in Ensembl or Yale Pseudogenes. Those which have any overlap spanning at least 80% of miRNA precursors annotated in miRBase or miRNA/tRNA/rRNA/snRNA/snoRNA annotated in Ensembl were also eliminated. The above rules apply in a strand-specific manner, thus allowing for calling antisense lncRNA.
3. Coding potential filter: Three open-reading-frames-related metrics were used to estimate whether the remaining transcripts exhibit significant protein-coding potential. The getorf program from the EMBOSS suite (33) was used to find ORFs in the candidates and therefore the length and coverage of ORFs and were calculated. ORFScan scores were calculated by the txCdsPredict program from UCSC. Transcripts with ORF <100 aa, ORF coverage less than 30% and txCdsPredict score <800 were classified as lncRNAs. Furthermore, we classified an additional subset of transcripts (ORF coverage between 30% and 90% and txCdsPredict score <800) that may include short ORFs and may serve as either lncRNAs or small peptides as TUCP (transcripts of uncertain coding potential), which was defined and used in several papers (15,34). The cutoff for these parameters was determined based on our survey of the known functional lncRNAs collected in the lncRNAdb database (5,35).
4. Known protein domains filter: Each remaining transcript was mapped to unitRef90 database with BlastX and transcripts with *E*-value < 1E–30 were removed. The resulting transcripts were translated in all three forward frames and fed to HMMER v3.0 (36) to examine the occurrence of any PfamA and PfamB known protein family domains collected in the Pfam database (37). Transcripts with a Pfam hit showing both the full sequence *E*-value < 1E–5 and the single best domain *E*-value < 1E–5 were removed.

All transcripts that passed all the filters above were classified based on their loci and treated as a set of lncRNAs in the following study. All the lncRNAs in deepBase v2.0 are named according to the following rules. The names of all the lncRNAs in deepBase v2.0 consist of three parts, ‘A-B-C’. Amongst, ‘A’ is composed by three letters, which represents the shorted name of species, ‘B’ is the gene ID and ‘C’ is the transcript ID. For example, hsa-lncRNA10932–8 means the 8th transcripts of gene lncRNA10932 in human.

Human, mouse and *C. elegans* circRNA genes were downloaded from circBase v1.0 (12) or obtained from the Supplementary Data of the original articles. In addition, we used the method ‘find_circ’ (16) to identify novel circRNAs from raw RNA-seq data of ENCODE and modENCODE. In brief, raw reads were first aligned to the genome. Then, unmapped sequencing reads were used for back-splice sites identification.

### Expression analysis of sRNAs and lncRNAs

DESeq (38) was used to evaluate the expression of small RNAs. FPKM (Fragments Per Kilobase of transcript per Million mapped reads) values were calculated by Cufflinks and used to evaluate the expression of lncRNAs. Cufflinks includes a program, Cuffnorm, which can be used to generate tables of expression values that are properly normalized for library size.

### Evolution analysis of long non-coding RNAs

The sequences of all identified long non-coding RNAs were extracted from genomes of corresponding species using Bedtools v2.17.0 (39). As described by Ulitsky et al. (21), we performed similarity searches with each lncRNA sequence in one species against that in the rest species using NCBI BLASTN with parameters ‘-task blastn -word_size 6 -evalue 1e–5 -strand plus’ and the resulting hits with the lowest *e*-value was kept as the most conserved lncRNA.

### Predicting the function of lncRNAs from co-expression networks

Expression correlation (Pearson correlation coefficient) between lncRNAs and protein-coding genes was estimated using coexpression program (the program is available from the authors upon request), which was written in C language and ALGLIB library, and p-value was adjusted with the False Discovery Rate (FDR) correction (40). GO ontology data for the Ensembl Genes was downloaded from the Ensembl website (41). There are thirteen functional categories for human genome. Enrichment analysis of these pathways in the dataset was determined using a hypergeometric test with Bonferroni and FDR correction (40).

## DATABASE CONTENT AND WEB INTERFACE

### The web-based exploration of sRNAs, lncRNAs and circRNAs

deepBase provides genome-wide identification of small RNAs in multiple types, including microRNAs, snoRNAs, rRNA-derived small RNAs. In total, there are 18 508 small RNAs are identified in 51 studies of 10 species (Table 1). Users who want to see the results of these small RNAs can use the Browse page. For example, deepBase v2.0 integrates a study which sequenced small RNAs in 60 samples of tumour or normal human cervical tissues (42). After our systematic re-annotation of all types of small RNAs in these samples, the ‘Browse’ page shows 3,176 small RNAs in the result table. By ordering the ‘Sample’ column of the table, users can find 14 small RNAs that could be detected in all of the 60 samples, including 12 miRNAs, one snoRNA (SNORD85) and one snRNA (RNU2–59P). By sorting the ‘Expression’ column of the table, the highest expressed miRNA hsa-mir-21 ranks on the top of the table. The ‘View’ button on the last column can link to the detail information of this miRNA, which displays the distribution of all the sequencing reads on the miRNA precursor in each sample. Most of the reads correspond to the mature arm of the miRNA precursor, and the highest one has 10 005 sequencing copies.

In the Browse page, we also provide integration of lncRNAs identified by RNA-seq, organized by different species and corresponding studies (Table 1). About half of the lncRNAs were not annotated in GENCODE (Supplementary Figure S1). For instance, we applied our lncRNA identification pipeline (detailed in Methods) to accurately annotate lncRNAs in mouse from RNA-seq data generated by a previous study (43). 14 462 mouse lncRNAs were identified, among which 11 220 were not annotated in GENCODE. We compared the result of lncRNAs in deepBase v2.0 with other three studies (13,15,34). Supplementary Table S2 shows the number of overlapped lncRNAs in deepBase v2.0 and novel lncRNAs that is not reported in the other studies. According to our results, although varied in amounts, the lncRNAs in different species shared common features (Supplementary Figures S2–S5). For example, the transcript length of lncRNAs is generally shorter than that of protein-coding genes but longer than other ncRNAs (Supplementary Figure S3). Users can view basic information of each lncRNA in the query result, and pressing the ‘Predict’ button of each entry will call the web-based lncFuntion program (introduced in the later session) to predict possible functions of the lncRNA.

### Expression profiles of sRNAs and lncRNAs

One of the most important features of miRNAs is their special expressions in different stages, tissues or cells. deepBase v2.0 provides the expression profiles of small RNAs in each sample. Users can find their interested miRNA name in the small RNA ‘Expression’ page. The heatmap will display the normalized expression number of miRNA in each sample. Click the name of the miRNA will link to the detail expression result page. Users can find that hsa-mir-122 is especially highly expressed in normal liver tissues compared to other samples.

Spatial and temporal specific expression is one of the well-characterized properties of lncRNAs (15). In the Expression page, expression profiles of lncRNAs are visualized through heatmaps, providing an easy way for users to spot tissue-specific lncRNAs. For instance, H19, a maternally imprinted lncRNA, was reported to be highly expressed in placenta and down-regulated in all tissues except skeletal muscle immediately after birth (44–46). This phenomenon can be observed through our expression heatmap of H19 generated from data of the Human Body 2.0 project (47).

### Evolutional conservation of lncRNAs in 14 species

We conducted evolution conservation analysis of lncRNAs in 14 species. Many studies show that most lncRNAs are lineage-specific and in fast phylogenetic evolution (13). Out of 18,964 lncRNAs in human we identified, 16 926 have at least one ortholog in primates. However, only 754 lncRNAs present to be conserved in Platypus. 13 800 human lncRNAs have not previously been annotated, among which hsa-lncRNA12238 from antisense is highly but only conserved in primates, indicative of weak evolution constraints. hsa-lncRNA12238 presents intensively high expression in human testis. To verify that, we then predict the function of hsa-lncRNA12238 in Function entry, and GO items show strong correlation with such reproductive process as spermatogenesis, sperm-egg recognition and so forth.

### lncSeeker web-server for the annotation and identification of lncRNAs

We provide a web-based tool named IncSeeker to implement our filtering pipeline of finding lncRNAs transcriptome assembled from RNA-seq data. Users can upload their transcriptome data in BED12 or GTF format, and our web-server will process the file by a series of tunable criterion and filter steps, including transcript length, exon number, coding ability and potential coding domain. A strict set of lncRNAs will then be displayed on the result page. To evaluate the performance of lncSeeker, we used lncSeeker to predict lncRNAs in GENCODE gene sets. There are 11 324 high-confidence lincRNA transcripts and 95 309 protein-coding transcripts in GENCODE Release 19 (GRCh37.p13). The result showed that the accuracy of lncSeeker was 96.82%, the sensitivity and specificity were 70.08% and 100% (Supplementary Table S3), respectively. We further compared lncSeeker with other four tools related with lncRNAs identification, getorf (33), txCdsPredict (48), CPAT (49) and CNCI (50). The result showed lncSeeker had the highest accuracy and specificity.

### lncFucntion web-server for the functional annotation of lncRNAs

A web-based tool, lncFunction, was also developed to predict lncRNA functions from coding and non-coding co-expression networks. There are six options on the query page, and the option ‘Input the Target LncRNA’ is required. The running parameters, expression values from selected study and every outcome in the three categories of function prediction are available for users to download.

## CONCLUSIONS

Although a few dozen of lncRNAs and circRNAs have been characterized to some extent and reported to function in important cellular processes (1–4), such as differentiation, development, proliferation, self-renewal, pluripotency, carcinogenesis and progression, the functions of most annotated ncRNAs are unknown (1–4). In this study, by analyzing a large set of sRNAs, lncRNAs and circRNAs identified from deep-sequencing datasets, we have annotated ncRNAs with a broad range of structural, expression, and evolutionary features.

Currently, there is an increasing amount of databases developed for ncRNAs, including NONCODE (51), lncRNAdisease (52), ncFANs (53), lncRNA2Function (54), FAME (55), miRo (56), miRGator (57) and lncRNAwiki (58). NONCODE (51) is a comprehensive database which contains all kinds of ncRNAs, but no evolution information for these ncRNAs. lncRNAdisease (52) is a specialized database which collects disease-associated lncRNAs and their interaction entries. On comparison with ncFANs (53), which is used to predict lncRNAs from ‘Affymetrix microarray datasets’, our tool is designed to identify lncRNAs from ‘RNA-Seq’ based datasets. Compared with other pipeline/tools (59), our pipeline integrates more tools as well as built-in scripts and annotates and identifies lncRNAs more reliably. Compared with our previous deepBase v1.0 which was designed to focus on small RNAs (60), deepBase v2.0 was designed to identify and annotate lncRNAs and circRNAs, and to predict the functions of lncRNAs. deepBase v2.0 also provides more comprehensive expression and evolution profiles of lncRNAs, circRNAs and small RNAs. The distinctive features of deepBase v2.0 include the following: (i) deepBase v2.0 has provided the most comprehensive expression analysis of sRNAs and lncRNAs from 1036 RNA-Seq datasets from 19 species. The constructed gene expression profiles of both ncRNAs and protein-coding genes from our analyses are a valuable resource for understanding the similarities and differences of transcriptional regulation of protein-coding genes and ncRNAs across different tissue/cell-line types. (ii) To the best of our knowledge, this is the first attempt to construct evolutional patterns of lncRNAs and circRNAs across several evolutional clades. Conservation patterns provided by our database may help biologists to select important ncRNAs for further functional validation. (iii) Two web-based tools, lncSeeker and lncFunction, can be used to identify high-confidence lncRNAs, and to predict lncRNA functions. We expect that access to these tools will enable more researchers to search for functions of novel lncRNAs in the ever-increasing amounts of deep-sequencing data.

## FUTURE DIRECTIONS

Next-generation sequencing technologies play vital roles in improving our understanding of functional genomics. As sRNA-Seq and RNA-Seq technology is applied to a broader set of species, cell lines, tissues and conditions, we will continually maintain and update the database. The integration of transcriptome datasets from the deepBase database, and the cancer genomics data and clinical information from the Gene Expression Omnibus (GEO), The Cancer Genome Atlas (TCGA) and International Cancer Genome Consortium (ICGC), will improve our understanding of expression and function of ncRNAs in diseases.

## AVAILABILITY

deepBase v2.0 is freely available at http://deepbase.sysu.edu.cn/ or http://biocenter.sysu.edu.cn/deepBase/. The deepBase data files can be downloaded and used in accordance with the GNU Public License and the license of primary data sources.

## References

[1] Guttman M., Rinn J.L. (2012). Modular regulatory principles of large non-coding RNAs. Nature.

[2] Ulitsky I., Bartel D.P. (2013). lincRNAs: genomics, evolution, and mechanisms. Cell.

[3] Bartel D.P. (2009). MicroRNAs: target recognition and regulatory functions. Cell.

[4] Jeck W.R., Sharpless N.E. (2014). Detecting and characterizing circular RNAs. Nat. Biotechnol..

[5] Amaral P.P., Clark M.B., Gascoigne D.K., Dinger M.E., Mattick J.S. (2011). lncRNAdb: a reference database for long noncoding RNAs. Nucleic Acids Res..

[6] Harrow J., Frankish A., Gonzalez J.M., Tapanari E., Diekhans M., Kokocinski F., Aken B.L., Barrell D., Zadissa A., Searle S. (2012). GENCODE: the reference human genome annotation for The ENCODE Project. Genome Res..

[7] Park C., Yu N., Choi I., Kim W., Lee S. (2014). lncRNAtor: a comprehensive resource for functional investigation of long non-coding RNAs. Bioinformatics.

[8] Yang J.H., Li J.H., Jiang S., Zhou H., Qu L.H. (2013). ChIPBase: a database for decoding the transcriptional regulation of long non-coding RNA and microRNA genes from ChIP-Seq data. Nucleic Acids Res..

[9] Bu D., Yu K., Sun S., Xie C., Skogerbo G., Miao R., Xiao H., Liao Q., Luo H., Zhao G. (2012). NONCODE v3.0: integrative annotation of long noncoding RNAs. Nucleic Acids Res..

[10] Kozomara A., Griffiths-Jones S. (2011). miRBase: integrating microRNA annotation and deep-sequencing data. Nucleic Acids Res..

[11] Li J.H., Liu S., Zhou H., Qu L.H., Yang J.H. (2014). starBase v2.0: decoding miRNA-ceRNA, miRNA-ncRNA and protein-RNA interaction networks from large-scale CLIP-Seq data. Nucleic Acids Res..

[12] Glazar P., Papavasileiou P., Rajewsky N. (2014). circBase: a database for circular RNAs. RNA.

[13] Necsulea A., Soumillon M., Warnefors M., Liechti A., Daish T., Zeller U., Baker J.C., Grutzner F., Kaessmann H. (2014). The evolution of lncRNA repertoires and expression patterns in tetrapods. Nature.

[14] Washietl S., Kellis M., Garber M. (2014). Evolutionary dynamics and tissue specificity of human long noncoding RNAs in six mammals. Genome Res..

[15] Cabili M.N., Trapnell C., Goff L., Koziol M., Tazon-Vega B., Regev A., Rinn J.L. (2011). Integrative annotation of human large intergenic noncoding RNAs reveals global properties and specific subclasses. Genes Dev..

[16] Memczak S., Jens M., Elefsinioti A., Torti F., Krueger J., Rybak A., Maier L., Mackowiak S.D., Gregersen L.H., Munschauer M. (2013). Circular RNAs are a large class of animal RNAs with regulatory potency. Nature.

[17] Hansen T.B., Jensen T.I., Clausen B.H., Bramsen J.B., Finsen B., Damgaard C.K., Kjems J. (2013). Natural RNA circles function as efficient microRNA sponges. Nature.

[18] Dinger M.E., Amaral P.P., Mercer T.R., Pang K.C., Bruce S.J., Gardiner B.B., Askarian-Amiri M.E., Ru K., Solda G., Simons C. (2008). Long noncoding RNAs in mouse embryonic stem cell pluripotency and differentiation. Genome Res..

[19] Young R.S., Marques A.C., Tibbit C., Haerty W., Bassett A.R., Liu J.L., Ponting C.P. (2012). Identification and properties of 1,119 candidate lincRNA loci in the Drosophila melanogaster genome. Genome Biol. Evol..

[20] Nam J.W., Bartel D.P. (2012). Long noncoding RNAs in C. elegans. Genome Res..

[21] Ulitsky I., Shkumatava A., Jan C.H., Sive H., Bartel D.P. (2011). Conserved function of lincRNAs in vertebrate embryonic development despite rapid sequence evolution. Cell.

[22] Pauli A., Valen E., Lin M.F., Garber M., Vastenhouw N.L., Levin J.Z., Fan L., Sandelin A., Rinn J.L., Regev A. (2012). Systematic identification of long noncoding RNAs expressed during zebrafish embryogenesis. Genome Res..

[23] Yang J.H., Shao P., Zhou H., Chen Y.Q., Qu L.H. (2010). deepBase: a database for deeply annotating and mining deep sequencing data. Nucleic Acids Res..

[24] Sayers E.W., Barrett T., Benson D.A., Bryant S.H., Canese K., Chetvernin V., Church D.M., DiCuccio M., Edgar R., Federhen S. (2009). Database resources of the National Center for Biotechnology Information. Nucleic Acids Res..

[25] Pruitt K.D., Tatusova T., Brown G.R., Maglott D.R. (2012). NCBI Reference Sequences (RefSeq): current status, new features and genome annotation policy. Nucleic Acids Res..

[26] Meyer L.R., Zweig A.S., Hinrichs A.S., Karolchik D., Kuhn R.M., Wong M., Sloan C.A., Rosenbloom K.R., Roe G., Rhead B. (2013). The UCSC Genome Browser database: extensions and updates 2013. Nucleic Acids Res..

[27] Harris T.W., Baran J., Bieri T., Cabunoc A., Chan J., Chen W.J., Davis P., Done J., Grove C., Howe K. (2014). WormBase 2014: new views of curated biology. Nucleic Acids Res..

[28] Tweedie S., Ashburner M., Falls K., Leyland P., McQuilton P., Marygold S., Millburn G., Osumi-Sutherland D., Schroeder A., Seal R. (2009). FlyBase: enhancing Drosophila Gene Ontology annotations. Nucleic Acids Res..

[29] Duan J., Li R.Q., Cheng D.J., Fan W., Zha X.F., Cheng T.C., Wu Y.Q., Wang J., Mita K., Xiang Z.H. (2010). SilkDB v2.0: a platform for silkworm (Bombyx mori) genome biology. Nucleic Acids Res..

[30] Kozomara A., Griffiths-Jones S. (2014). miRBase: annotating high confidence microRNAs using deep sequencing data. Nucleic Acids Res..

[31] Kim D., Pertea G., Trapnell C., Pimentel H., Kelley R., Salzberg S.L. (2013). TopHat2: accurate alignment of transcriptomes in the presence of insertions, deletions and gene fusions. Genome Biol..

[32] Trapnell C., Hendrickson D.G., Sauvageau M., Goff L., Rinn J.L., Pachter L. (2013). Differential analysis of gene regulation at transcript resolution with RNA-seq. Nat. Biotechnol..

[33] Rice P., Longden I., Bleasby A. (2000). EMBOSS: the European Molecular Biology Open Software Suite. Trends Genet..

[34] Iyer M.K., Niknafs Y.S., Malik R., Singhal U., Sahu A., Hosono Y., Barrette T.R., Prensner J.R., Evans J.R., Zhao S. (2015). The landscape of long noncoding RNAs in the human transcriptome. Nat. Genet..

[35] Quek X.C., Thomson D.W., Maag J.L., Bartonicek N., Signal B., Clark M.B., Gloss B.S., Dinger M.E. (2015). lncRNAdb v2.0: expanding the reference database for functional long noncoding RNAs. Nucleic Acids Res..

[36] Finn R.D., Clements J., Eddy S.R. (2011). HMMER web server: interactive sequence similarity searching. Nucleic Acids Res..

[37] Finn R.D., Bateman A., Clements J., Coggill P., Eberhardt R.Y., Eddy S.R., Heger A., Hetherington K., Holm L., Mistry J. (2014). Pfam: the protein families database. Nucleic Acids Res..

[38] Anders S., Huber W. (2010). Differential expression analysis for sequence count data. Genome Biol..

[39] Quinlan A.R., Hall I.M. (2010). BEDTools: a flexible suite of utilities for comparing genomic features. Bioinformatics.

[40] Benjamini Y., Hochberg Y. (1995). Controlling the false discovery rate: a practical and powerful approach to multiple testing. J. R. Stat. Soc. Ser. B (Methodological).

[41] Flicek P., Ahmed I., Amode M.R., Barrell D., Beal K., Brent S., Carvalho-Silva D., Clapham P., Coates G., Fairley S. (2013). Ensembl 2013. Nucleic Acids Res..

[42] Witten D., Tibshirani R., Gu S.G., Fire A., Lui W.O. (2010). Ultra-high throughput sequencing-based small RNA discovery and discrete statistical biomarker analysis in a collection of cervical tumours and matched controls. BMC Biol..

[43] Merkin J., Russell C., Chen P., Burge C.B. (2012). Evolutionary dynamics of gene and isoform regulation in Mammalian tissues. Science.

[44] Brunkow M.E., Tilghman S.M. (1991). Ectopic expression of the H19 gene in mice causes prenatal lethality. Genes Dev..

[45] Arima T., Matsuda T., Takagi N., Wake N. (1997). Association of IGF2 and H19 imprinting with choriocarcinoma development. Cancer Genet. Cytogenet..

[46] Banet G., Bibi O., Matouk I., Ayesh S., Laster M., Kimber K.M., Tykocinski M., de Groot N., Hochberg A., Ohana P. (2000). Characterization of human and mouse H19 regulatory sequences. Mol. Biol. Rep..

[47] Farrell C.M., O'Leary N.A., Harte R.A., Loveland J.E., Wilming L.G., Wallin C., Diekhans M., Barrell D., Searle S.M., Aken B. (2014). Current status and new features of the Consensus Coding Sequence database. Nucleic Acids Res..

[48] Kent W.J., Sugnet C.W., Furey T.S., Roskin K.M., Pringle T.H., Zahler A.M., Haussler D. (2002). The human genome browser at UCSC. Genome Res..

[49] Wang L., Park H.J., Dasari S., Wang S.Q., Kocher J.P., Li W. (2013). CPAT: Coding-Potential Assessment Tool using an alignment-free logistic regression model. Nucleic Acids Res..

[50] Sun L., Luo H.T., Bu D.C., Zhao G.G., Yu K.T., Zhang C.H., Liu Y.N., Chen R.S., Zhao Y. (2013). Utilizing sequence intrinsic composition to classify protein-coding and long non-coding transcripts. Nucleic Acids Res..

[51] Xie C., Yuan J., Li H., Li M., Zhao G., Bu D., Zhu W., Wu W., Chen R., Zhao Y. (2014). NONCODEv4: exploring the world of long non-coding RNA genes. Nucleic Acids Res..

[52] Chen G., Wang Z., Wang D., Qiu C., Liu M., Chen X., Zhang Q., Yan G., Cui Q. (2013). LncRNADisease: a database for long-non-coding RNA-associated diseases. Nucleic Acids Res..

[53] Liao Q., Xiao H., Bu D., Xie C., Miao R., Luo H., Zhao G., Yu K., Zhao H., Skogerbo G. (2011). ncFANs: a web server for functional annotation of long non-coding RNAs. Nucleic Acids Res..

[54] Jiang Q., Ma R., Wang J., Wu X., Jin S., Peng J., Tan R., Zhang T., Li Y., Wang Y. (2015). LncRNA2Function: a comprehensive resource for functional investigation of human lncRNAs based on RNA-seq data. BMC Genomics.

[55] Ulitsky I., Laurent L.C., Shamir R. (2010). Towards computational prediction of microRNA function and activity. Nucleic Acids Res..

[56] Lagana A., Forte S., Giudice A., Arena M.R., Puglisi P.L., Giugno R., Pulvirenti A., Shasha D., Ferro A. (2009). miRo: a miRNA knowledge base. Database-Oxford.

[57] Cho S., Jang I., Jun Y., Yoon S., Ko M., Kwon Y., Choi I., Chang H., Ryu D., Lee B. (2013). miRGator v3.0: a microRNA portal for deep sequencing, expression profiling and mRNA targeting. Nucleic Acids Res..

[58] Ma L.N., Li A., Zou D., Xu X.J., Xia L., Yu J., Bajic V.B., Zhang Z. (2015). LncRNAWiki: harnessing community knowledge in collaborative curation of human long non-coding RNAs. Nucleic Acids Res..

[59] Ilott N.E., Ponting C.P. (2013). Predicting long non-coding RNAs using RNA sequencing. Methods.

[60] Yang J.H., Shao P., Zhou H., Chen Y.Q., Qu L.H. (2010). deepBase: a database for deeply annotating and mining deep sequencing data. Nucleic Acids Res..

